# Rapid Generation and Detection of Biomimetic Oxygen Concentration Gradients In Vitro

**DOI:** 10.1038/s41598-017-13886-z

**Published:** 2017-10-18

**Authors:** Daud H. Khan, Steven A. Roberts, John Robert Cressman, Nitin Agrawal

**Affiliations:** 1 Bioengineering Department, George Mason University, Fairfax, 22030 USA; 2 School of Physics, George Mason University, Fairfax, 22030 USA

## Abstract

Hypoxic regions exist within most solid tumors and often lead to altered cellular metabolism, metastasis, and drug resistance. Reliable generation and detection of biomimetic gaseous gradients *in vitro* is challenging due to low spatiotemporal resolution and poor longevity of gradients utilizing microfluidic techniques. Here, we present a novel and simplistic approach for producing gradients of dissolved oxygen (DO) within a lab-on-a-chip platform. Linear and stable DO gradients with high spatial resolution are established by introducing pre-gassed media into the gradient generating network. An underlying platinum(ii) octaethlporphyrin ketone (PtOEPK) based sensor layer allows parallel detection of oxygen. A thin 3-sided glass coating on the inner channel walls prevents multi-directional diffusion of ambient oxygen across PDMS preserving the gradient resolution and stability. Viability analysis of normal mammary epithelial cells (MCF-12A) under oxygen gradients revealed 70% mortality after 6 hours of hypoxic exposure. Biological applicability of the platform was further validated by demonstrating increase in endoplasmic reticulum stress of MDA-MB-468 cells with time and with increasing oxygen tension. The unique ability to establish parallel or opposing gradients of gases and chemicals offers the potential for a wide range of applications in therapeutic development, and fundamental understanding of cellular behavior during hypoxia.

## Introduction

Oxygen homeostasis is critical for the existence of multicellular organisms. While oxygen deficiency or hypoxia can result in pathological conditions such as ischemia and tumorigenesis^1–3^, elevated oxygen levels (hyperopia) can lead to generation of reactive oxygen species and free-radicals^4^. During cancer progression, rapid growth of tumor lesions lead to internal hypoxic conditions, which in turn initiates angiogenesis, followed by tumor cell invasion into healthy tissues and eventual metastasis^3,5–8^. Blood vessels generated from pathological angiogenesis are highly disorganized and morphologically irregular, impeding nutrient supply and metabolite clearance^9–11^ and eventually culminating in acquiring drug resistance^12^.

*In vitro* generation as well as detection of dissolved oxygen (DO) is crucial for a wide range of applications including therapeutic development and understanding of disease functions. Typically, exploration of cellular functions under controlled oxygen environments are conducted using specialized culture chambers (e.g. O_2_ incubators)^13^. However, such systems can only maintain a singular oxygen concentration, significantly varying from actual physiological conditions where DO gradients normally exist within tissues^14,15^. To address this, bi-compartmental open-well cell culture devices, similar to Boyden chambers, have been developed where oxygen (and nitrogen) flowing in the lower compartment diffuses through a polydimethylsiloxane (PDMS) barrier into the upper compartment where the cells are seeded^16^. The introduction and evolution of microfluidic devices have further improved this scenario, offering the ability to rapidly generate a range of oxygen tension profiles *in vitro* and investigate cellular responses under oxygen gradients^17–19^. These utilize either multiple gas inlets to establish DO gradients or chemical quenchers such as Na_2_SO_3_ that deplete oxygen from the media^20–23^. However, the requirement of continuous gas flow not only introduces potential risks of bubble formation and media evaporation, but also limits the portability of experimental setups as they are confined to the proximity of compressed gas tanks. On the other hand, chemical quenchers are cytotoxic and are often undesirable while investigating therapeutic effects of drugs under hypoxic conditions^24,25^. Both approaches are only able to generate DO gradients with low-to-moderate spatial resolution. Increasing the number of gas inlets may improve the spatial resolution, but with aforementioned risks and disadvantages involved.

In addition to gradient generation, the ability to detect local DO levels at any given time is critical to validate both reproducibility and overall experimental outcomes. Detection of DO is achieved either electrochemically through amperometric Clark-type electrodes or optically via oxygen-sensitive dyes^18^. Though extensively used in commercial gas detectors^26,27^, the macroscale size of the electrode, along with low sensitivity, long response time, and analyte depletion and fouling by organic fluids^28^ restricts its usage in microfluidic oxygen detection. Optical-based detection utilizes a polymer-encapsulated luminescent dye, such as phosphorescent metalloporphyrin complexes and fluorescent organo-ruthenium complexes^29,30^. DO content is proportional to the quenching of dye luminescence, thus enabling non-invasive and dynamic sensing of oxygen. Although nanoparticles incorporating such dyes have recently been synthesized, potential leaching and subsequent cytotoxicity issues have limited their adaptation for biological applications^31,32^.

**Figure 1:**
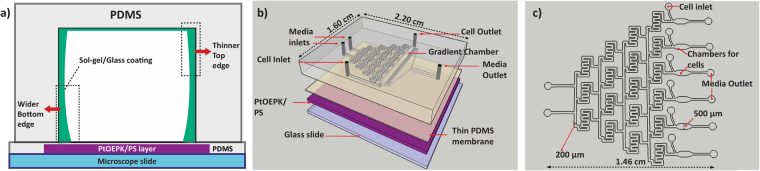
(**a**) Cross-section schematic of glass coated channel showing placement of different layers. As the device is heated from the bottom by placement on a hot plate, coating thickness is higher at the bottom due to temperature gradient across the height of channel that affects sol-gel reaction rate; (**b**) 3D layout of the single-outlet device illustrating the bottom PtOEPK sensor layer in purple, followed by the thin PDMS film and a top transparent PDMS layer containing the channel network; (**c**) 2D layout of the multiple-outlet device design.

Herein, we have developed a novel and simplistic technique of generating and detecting stable biomimetic oxygen gradients with high spatial resolution. Simultaneous infusion of O_2_-rich and O_2_-depleted media (pre-gassed) allows generation of stable DO gradients by simply utilizing the microfluidic split-and-recombine strategy, as seen with chemical gradients^33,34^. A 3-sided glass-like coating within the PDMS microchannel prevents multi-directional diffusion of oxygen and maintains a spatially as well as temporally stable gradient. Thus, the requirement of gas inlets and chemical quenchers is eradicated. Real-time detection of DO is achieved by a layer of platinum(ii)octaethylporphyrin ketone embedded in a polystyrene matrix (PtOEPK/PS) underlying the gradient channel (Fig. 1a). A thin PDMS membrane separates the sensor-layer from cell chamber to prevent any dye mediated toxicity. Computational modeling using COMSOL Multiphysics software was performed to verify gradient formation and device optimization. Two different designs have been developed, suitable for specific applications. First, gradients were established inside a single-outlet device, similar to the microfluidic devices first established by Jeon and colleagues^35^ to generate intrinsic chemical gradients, where all the channels (250 μm wide) unite and converge into a single outlet (Fig. 1b). To further broaden the versatility of the gradient system, a multiple-outlet device was designed (Fig. 1c). Here, the individual concentration streams remain separated to explore the possibility of forming unit concentrations of DO in each outlet. Among numerous potential applications, proof-of-concept functionality of these platforms was demonstrated through evaluation of ER stress of metastatic breast cancer cells (MDA-MB-468) under gradients from 0–21% dissolved oxygen in the single-outlet device and viability analysis of MCF-12A (immortalized normal mammary epithelial cells) in the multiple-outlet device. To our knowledge, no other existing approach offers such convenience and versatility to establish a wide range of gaseous gradients under biomimetic conditions. Our proposed platform and the unique strategy underlying gradient generation provides a novel, feasible, and reproducible way to study cell functions under hypoxic or hyperoxic conditions.

## Results

### Deposition and characterization of gas impermeable sol-gel coating

**Figure 2:**
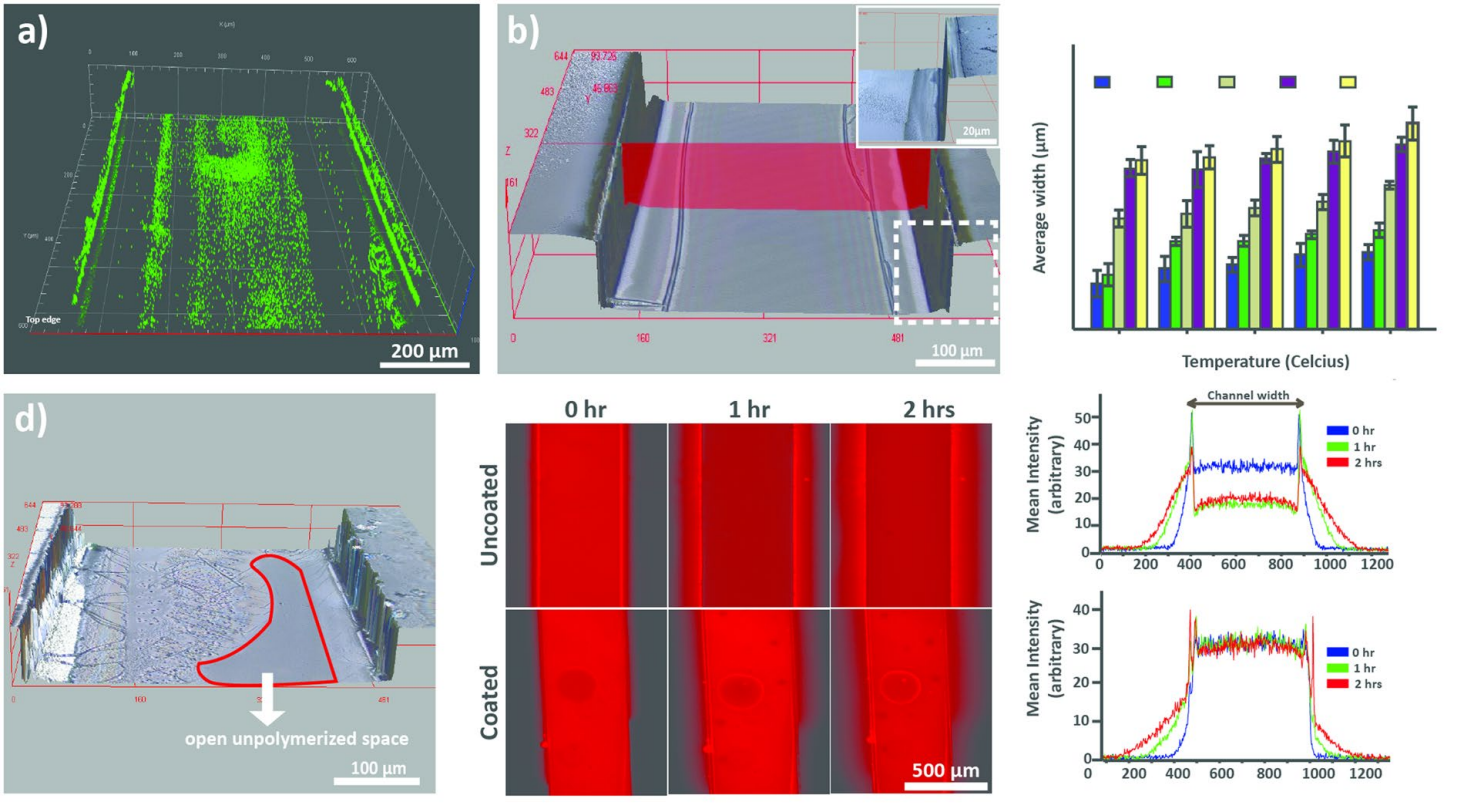
(**a**) Z-stack fluorescent image of the inverted channel with 3-sided glass coating. 1 μm fluorescent polystyrene beads were mixed in the sol-gel solution prior to coating formation to facilitate imaging the presence of glass coating (**b**) High resolution 3D phase-contrast image of the 3-sided coated channel at optimal conditions showing glass-coating (~7 μm thick) on walls with no unpolymerized spaces and minimal crack formation. The image was taken with an Olympus laser confocal surface profilometer (**c**) Variation in average glass coating thickness across different times and temperatures and (**d**) Glass coating with unpolymerized spaces, typical at lower temperatures and reaction times (**e**) Images and corresponding mean intensity curves showing leaching profile comparison of coated versus uncoated channels using 1 μM rhodamine B solution in DI water.

A modification of known sol-gel chemistry was used to deposit the 3-sided silicon dioxide polymer within the channels (See Methods). The polymerization process consists of 3 main steps; first the hydrolysis of precursor alkoxysilane groups into silanol groups by ethanol, followed by oligomerization of silanol monomers in the presence of an acid^36^. The silanol oligomers then form hydrogen bonds with hydroxyl groups present on the walls of plasma-treated PDMS subtrate^36^. Finally heat is applied to remove H_2_O molecules and form covalent oxide bonds, creating the polymerized glass layer^36^. Two key parameters, temperature and reaction time, were characterized to achieve optimal and reproducible coatings^36,37^. Varying temperatures from 60–100 ^◦^C (10 ^◦^C increments) and reaction times from 20–100 s (20 s increments) were investigated. To verify the formation of glass coatings, two independent methods were utilized. Initially, we dissolved fluorescent polystyrene beads into the sol-gel solution at a ratio of 1:10 (v/v) prior to the coating formation. Imaging through fluorescent microscopy not only confirmed the presence of a three-sided glass coating but also facilitated measurements of coating dimensions (Fig. 2a). This was further validated by imaging the coated channel with a confocal surface profilometer at high resolution (Fig. 2b).

As expected, the average thickness of cured glass-coating increased progressively with both temperature and time ranging from approximately 3–15 µm (Fig. 2c, Table S1). We observed that the coating thickness increased exponentially with increasing times while following a linear pattern with increasing temperatures. Unpolymerized spaces were more prominent at lower temperatures and for shorter time spans (Fig. 2d) whereas the frequency of cracked glass coatings increased significantly with increasing time or temperature (Table S1). We believe the crack formation results from non-uniform cooling effect when the unpolymerized sol-gel is flushed out using compressed air. The crack formation was significantly more noticeable beyond a coating thickness of approximately 10 µm. For creating a diffusion barrier and for the specific channel dimensions of our device design, heating at 80 ^◦^C for 40 s was identified as an optimal condition that produced glass coating thicknesses of 6.8 ± 0.79 µm with no unpolymerized open spaces and lower frequency of cracks. These conditions were used for all subsequent experiments. We also observed that due to thermal gradient along the height of PDMS device from the hot plate, an arch-like coating was created, i.e. a slightly wider film (by ≈2 µm) at the lower region of side walls as compared to the top. However, this had no effect on inhibiting gaseous diffusion across the barrier.

The impermeability of the coating was verified by leaching experiment with rhodamine B solution, which is known to diffuse into the PDMS. The average intensity of 1 µM rhodamine B solution within the channels was reduced by approximately 50% of the original fluorescence over a period of 2 hours for uncoated channels and significant diffusion of dye outside the channel was observed (Fig. 2e). Whereas, no leaching was observed for glass coated channels and the fluorescence intensity remained unchanged. The two peaks in the mean intensity curve represent accumulated rhodamine B solution on the sides of channel wall.

### Integration and calibration of oxygen sensitive PtOEPK layer

**Figure 3:**
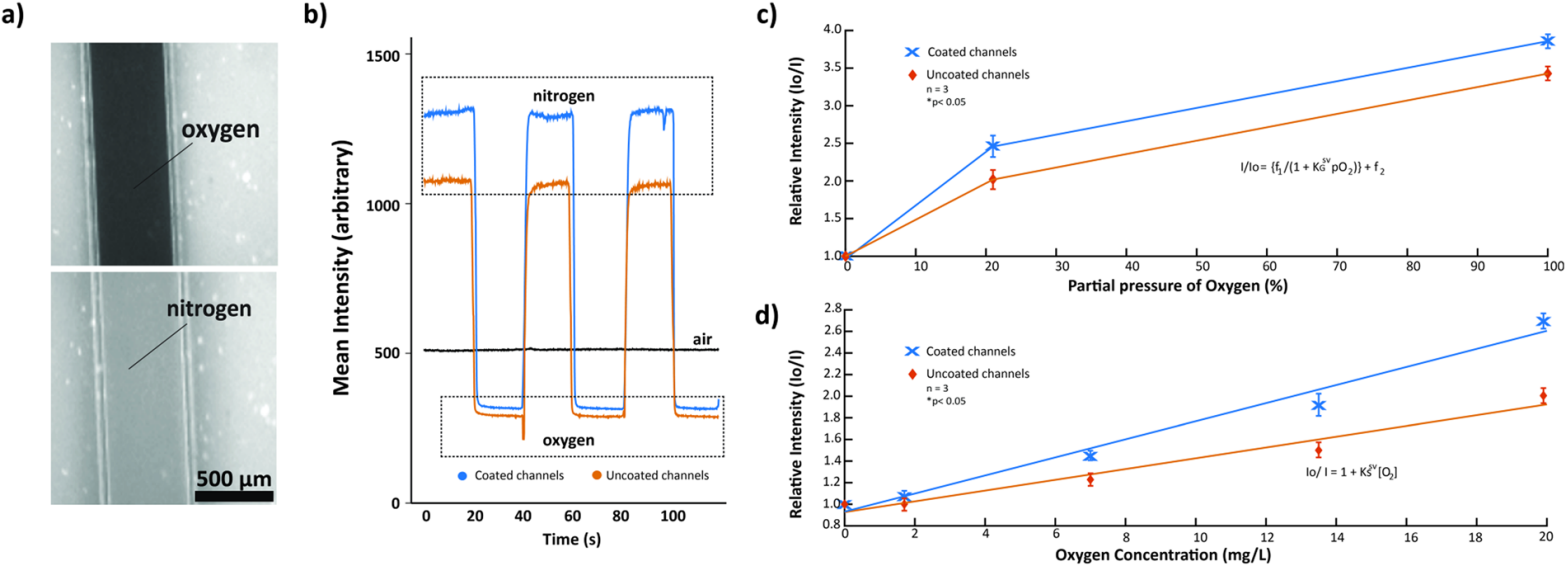
(**a**) Change in intensity of the underlying PtOEPK dye when exposed to gaseous nitrogen (top) and oxygen (bottom) gas. Gases were introduced into the channel by connecting regulated compressed tanks of respective gases through tygon tubing (**b**) Dynamic sensing ability and comparison of coated versus uncoated channels (air being considered baseline) after periodic introduction of gaseous oxygen and nitrogen. (**c**) Calibration curve for gaseous oxygen detection of coated and uncoated channels. (**d**) Calibration curve for dissolved oxygen detection illustrating the relative chemiluminescent intensity of PtOEPK corresponding to different concentrations of dissolved oxygen in DI water.

A 1.3 µm thin film of 7% w/w PtOEPK/PS matrix was spin-coated on a 1 × 3 inch microscope slide to form the sensor layer^18^. Although spin-coating at lower speeds increases the thickness and PtOEPK molecules available for oxygen quenching, it significantly reduces the spreading area of the sensor layer, preventing gradient detection and profiling across the desired regions of both device designs. The spin-coating conditions and concentrations of dye (see Methods) were optimized to create PtOEPK/PS films with maximum spreading area and uniform thickness (verified with confocal surface profilometer). To prevent possible leaching and cytotoxicity by PtOEPK, when in direct contact with cells^38^, an additional 1.5 µm (±0.2) thin layer of PDMS was spin-coated on top of the sensor layer. This creates a barrier between cells and PtOEPK while still allowing dissolved oxygen to quickly diffuse through the barrier and interact with underling PtOEPK. Oxygen diffuses nearly twice as fast through PDMS than water^39^. Thus, rapid vertical diffusion of oxygen through thin PDMS film and subsequent dye quenching does not affect the signal intensity of detected gradients. To perform the calibration of PtOEPK film, a simple straight channel PDMS device was plasma bonded on top of the composite sensor/PDMS layers and pure nitrogen and oxygen gases were introduced successively. Introduction of pure gaseous oxygen resulted in an immediate decrease in the emitted dye luminescence and vice versa for nitrogen (Fig. 3a). Response data for both coated and uncoated channels show an almost immediate change in luminescence upon alternating the gases, the relative change being superior for coated channels (Fig. 3b). Gaseous calibration data is coherent with the non-linear Stern-Volmer relationship previously described in literature using metalloporphyrin dyes^40^. The coated channels demonstrated superior intensity ratio I_0_/I_100_ of 3.85 and an improved sensitivity by 20% compared to uncoated channels (Fig. 3c). The non-linearity results from the fraction of dye molecules that remain unquenched^40^.

Subsequently, primary DO calibration was performed by using DI water of varying oxygen concentrations. No recognizable differences were observed in the DO levels for DI water, phosphate-buffer saline (PBS), or the cell culture media for similar bubbling durations (data not shown). Thus, calibration was performed using DI water to avoid fouling of the sensor probe. To account for photobleaching of the PtOEPK dye, calibration was performed every time prior to the experimentation. Maximum DO detection was restricted to 19.9 mg/L due to the detection limit of polarographic meter used in our experiments (Milwaukee MW600) and intermediate DO levels were acquired by proportionately mixing 0.1 and 19.9 mg/L DI water. This was sufficient to yield a broad and satisfactory range of DO levels, from hypoxic to hyperoxic conditions (0.1 1.7, 7.0, 13.5 and 19.9 mg/L). For reference, atmospheric conditions with 21% partial pressure of oxygen corresponds to 7.8 mg/L of DO levels in aqueous solutions. Measurements of DO were taken simultaneously during gas bubbling and mixing. The sensor probe utilized here is incapable of taking measurements beyond 19.9 mg/L and provides an error message, thus enabling us to determine exact gas bubbling durations. Plots of relative intensity versus dissolved O_2_ concentration followed a linear Stern-Volmer trend, with coated devices demonstrating superiority over the uncoated ones (p < 0.05) (Fig. 3d). Since luminescence intensity depends on the number of PtOEPK molecules available to interact with diffusing oxygen, calibration was performed for each device independently prior to its utilization to compensate for photobleaching effects. These results indicate that the integrated PtOEPK sensor layer can be utilized for real-time sensing of dissolved oxygen and the glass coated devices offer significantly improved detection sensitivity as compared to bare PDMS devices.

### Simulation, characterization and generation of oxygen gradients

**Figure 4:**
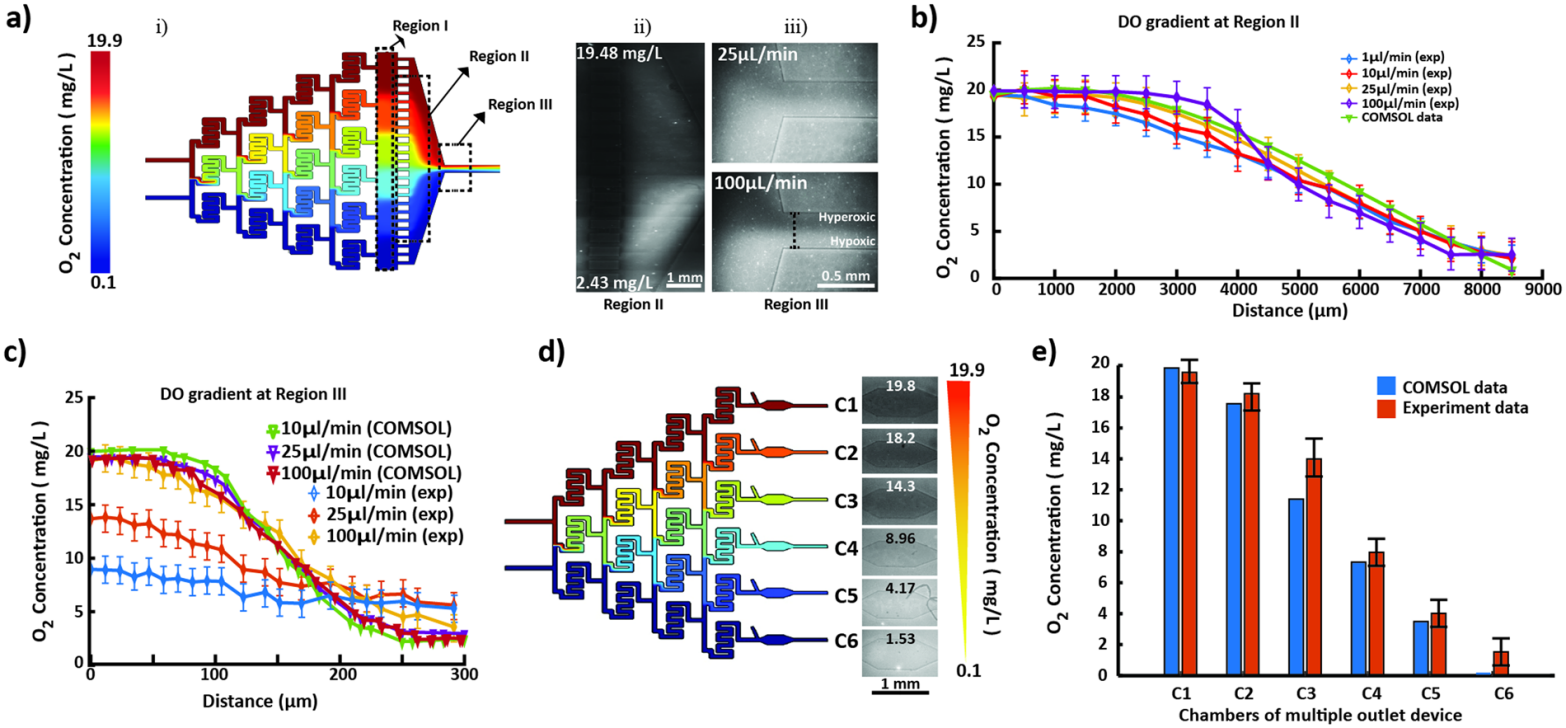
(**a**) (i) COMSOL simulated oxygen gradient profile in single-outlet device at 1 μL/min, (ii) Oxygen concentration gradient across region II detected by PtOEPK luminescence. The profile of the left edge of region II which is parallel to Region I was analyzed. The gradient was established by infusing 19.9 mg/L and 0.1 mg/L oxygenated water at 10 μL/min flow rate. (iii) Profile of oxygen concentration gradient across the outlet or region III, showing increasing slope of gradient with increasing flow rates (flowing 19.9 mg/L and 0.1 mg/L DO water at 25 μL/min and 100 μL/min). (**b**) Concentration plot comparison of COMSOL and experimental data across the single-outlet at different flow rates in region II; (**c**) Concentration plot illustrating progressive trend in the slope of gradient across the outlet or region III with respect to the flow rate and comparison with COMSOL data. (**d**) Unit concentrations of oxygen formed in each chamber of the multiple-outlet device (both COMSOL and experimental) at 10 μL/min flow rate. (**e**) Comparison of actual versus predicted (COMSOL) concentrations within individual chambers of the multiple-outlet device at 10 μL/min flow rate.

For convenience, specific areas of the channel network, where oxygen gradients are generated, are designated as regions I, II and III. Region I and II comprise the gradient chamber while region III is the outlet. The flow-rates required to produce a linear, working gradient were initially determined using COMSOL simulation for both designs. Taking the diffusivity of oxygen in water into consideration along with incompressible flow and no-slip and non-diffusive boundary conditions, oxygen gradients were simulated at a minimum flow rate of 10 nL/min (Figure S3). Non-diffusive boundary conditions were assumed to reflect the relative impermeability of silicon oxide polymer coating. The diffusion co-efficient of oxygen in PDMS at 298.15 K or room temperature is approximately 1.7 times greater than water (3.55 × 10^−5^ cm^2^/s vs. 2.05 × 10^−5^ cm^2^/s)^39^. Therefore, the coating provides the necessary non-diffusive boundary without which, net oxygen would diffuse across PDMS walls significantly faster as compared to its lateral diffusion across the aqueous streamlines. Hence at steady state, the concentration of DO at any given coordinate of the microfluidic network is constant proving a continuous, stable and linear gradient. In case of the single-outlet device, though the overall slope of gradient across region I was constant (2.19 *10^−3^ mg L^−1^μm^−1^) irrespective of the flow rate, the long residence time at the minimum flow rate of 10 nL/min caused the gradient to diminish and completely equilibrate by the time it reached the outlet (Figure S3). Increasing the flow rate to 1 μL/min significantly improved stability of the gradient (Fig. 4a (i)). The equilibration issue was not observed for the multiple-outlet design as there is no convergence point and each intermediate concentration stream remains discrete. Beyond 100 μL/min, diffusion of oxygen within both single- and multiple-outlet devices was dominated by the flow rate such that higher flow rates did not allow sufficient residence times for the diffusion mediated mixing to occur within each split channel affecting the linearity of gradient.

For the given concentration of PtOEPK molecules present under the flow channels, a minimum flow rate of 1 µL/min was required to produce an optically detectable luminescence. Interestingly, the overall slope of experimentally generated gradient across regions I and II in the single-outlet device at different flow rates (1–100 µL/min) was constant and comparable with the COMSOL data, with a slightly diminished value of 2.04 * 10^−3^ mg L^−1^μm^−1^ (Fig. 4b). One-way ANOVA analysis illustrated that this difference was statistically insignificant (p > 0.05). However, the high diffusivity of oxygen in DI water, and relatively long residence times due to large width in regions I and II allow gradients to equilibrate within these regions. At the lower range of flow rates (1–25 µL/min), the equilibration effects are exacerbated by the time streamlines converge in the outlet and thus diminished gradients were observed in region III (measured across the two edges of the outlet) as compared to COMSOL. The variability between experimental and COMSOL data is insignificant at higher flow rate (e.g. 100 µL/min, Fig. 4c) likely due to faster replenishment of oxygen molecules at steady state resulting in a more discrete detection of PtOEPK quenching. Loss of oxygen through various cracks across the glass coating could also contribute towards weak signals at lower flow rates. As such, the most visually prominent concentration profile at region III was produced at 100 µL/min (Fig. 4 a (iii),4c). The equilibration issue at lower flow rates can be easily resolved by appropriately altering channel dimensions to obtain higher linear flow velocities. Similar trends were observed when experimentation was repeated in PBS as well as cell culture media suggesting that the ability to hold dissolved oxygen was not affected by the choice of aqueous solutions.

Time-based experiments revealed that the intrinsic DO gradient generated within regions I and II was stable for at least 60 minutes (after which monitoring was stopped) but can be maintained for prolonged periods (Figure S4). In case of multiple-outlet device/design, slightly smaller dimensions (200 µm wide channels and 1.46 cm in total length) and physically separated outlets created and confined specific DO concentrations within each chamber of the device (corresponding to the intermediate concentration regimes created by the split channel network). The shortened length reduced the residence time for each flow rate and an improved spatial resolution was observed (Fig. 4d) with only slight variations between experimental and COMSOL data at various flow rates (Fig. 4e).

### Evaluation of ER stress under gradients of oxygen

**Figure 5:**
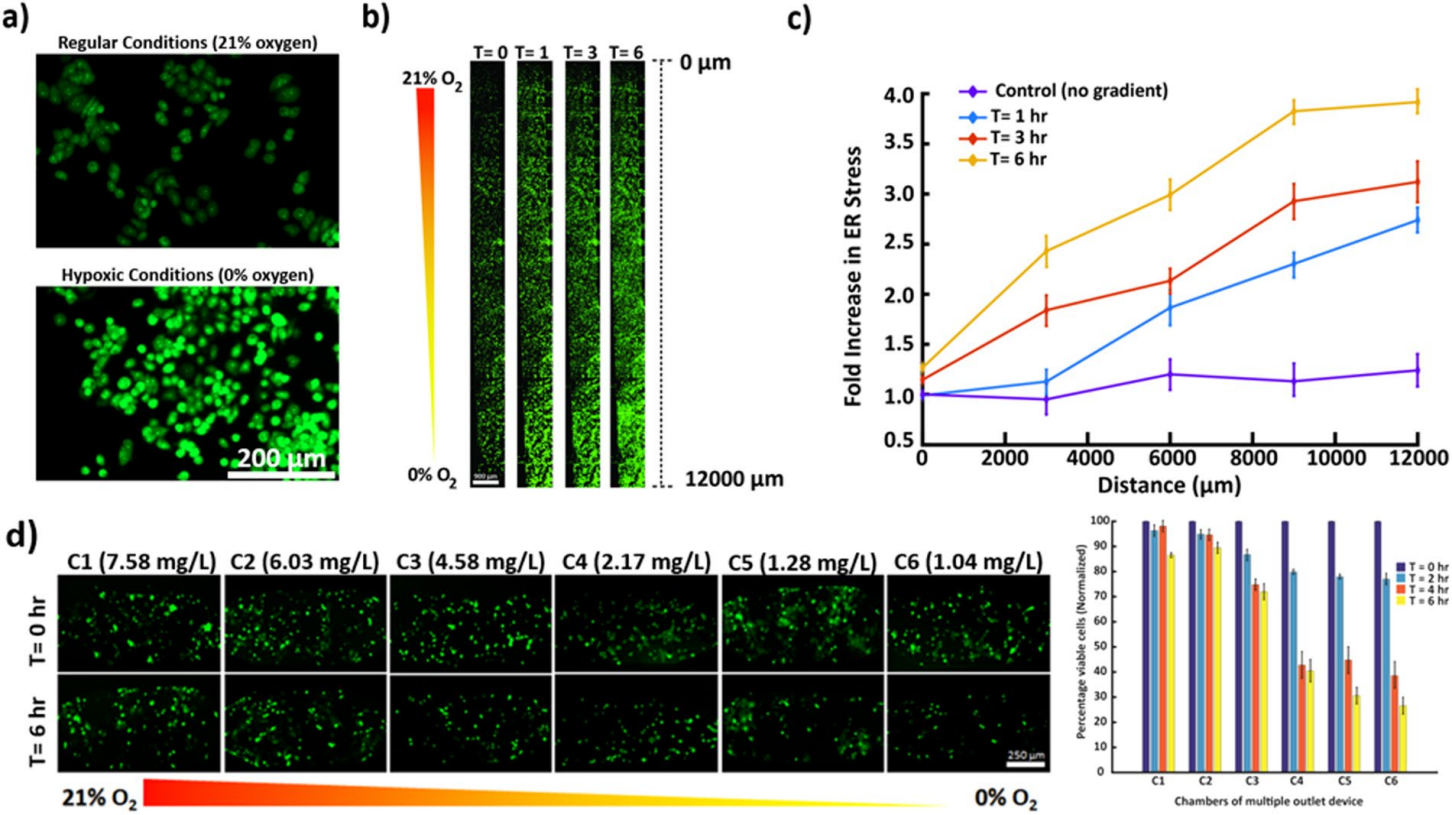
(**a**) Comparison of ER stress of MDA-MB-468 cells via Thioflavin T fluorescence under normal and hypoxic conditions, showing elevated ER stress levels in hypoxia. (**b**) ER stress across region I in single-outlet deice with oxygen gradient generated at a flow rate of 1 μL/min at 0 hr, 1 hr, 3 hr and 6 hr. The fluorescence increases under oxidative stress resulting in progressively higher fluorescence towards the hypoxic edge of region I of the single-outlet device and with time. (**c**) Plot showing fluorescence trend of MDA-MB-468 cells across region I, illustrating gradual and significant increase in ER stress from baseline due to oxidative stress at different time periods. Nearly a 4-fold increase is noticeable in the most hypoxic region (**d**) Viability analysis of MCF-12A under hypoxia gradient established within the multiple-outlet device. Incubating cells for 6 hours under oxygen gradient illustrated increasing mortality with reducing oxygen levels. The concentration values labelled for each panel were experimentally derived.

Hypoxia is known to induce unfolded protein response and increased endoplasmic reticulum (ER) stress in many cell types including the breast epithelial cell line MDA-MB-468. To explore the effects of hypoxic gradients on ER stress of these cells and to evaluate the overall applicability of the microfluidic hypoxia platform, we cultured MDA-MB-468 cells within region I and exposed them to a gradient of oxygen from 0–21%. Thioflavin T (indicator of ER stress) induced fluorescence linearly increased with increasing levels of hypoxia across the width of region I (Fig. 5a,b) with a 4-fold increase from baseline (t = 0 hour) fluorescence after 6 hours in the most hypoxic location (Fig. 5c). Time-lapse imaging also indicated a gradual but consistent increase in fluorescence intensity throughout region I. MDA-MB-468 cells incubated in a separate device underwent the identical procedure with the absence of an oxygen gradient yielding a relatively constant fluorescence of thioflavin T throughout region I (Fig. 5c). The observations were coherent with our control experiments conducted in well-plates and with the literature^41^. These results demonstrate potential applicability of the proposed hypoxia gradient generation strategy for a variety of real-time biological studies.

### Viability analysis of mammary epithelial cells MCF-12A under gradients of oxygen

To demonstrate a proof-of-concept application of the multiple-outlet device, cell viability analysis was performed at different hypoxic levels utilizing a normal mammary epithelial cell line (MCF-12A). Cells were cultured in each outlet chamber (C1-C6) for viability analysis while a 96-well plate setup maintained within the hypoxia incubation chamber (95% nitrogen & 5% CO_2_) was used as control. Following a 6 hour exposure to oxygen gradient (0–21% in C1 through C6 respectively), a decline in cell viability was observed across individual chambers with time. A drastic reduction in viability was observed in chambers C4-C6 with approximately 60–70% mortality respectively as compared to chambers C1-C3 (12–30% respectively). Similar results were observed in time-based analysis of cell viability where, a sharp decline was observed after 2 hours of hypoxic exposure in chambers C4-C6 (Fig. 5d). DMEM-F12 contains relatively high levels of glucose (3.15 g/l, 17.5 mM), which is enough to maintain viability of immortalized cells in low oxygen. However, oxygen concentrations below a critical level of approximately 5% can alter physiological functions of normal cells and diminish glucose uptake^42^. Chambers C4, C5, and C6 correspond to oxygen levels of 3.36%, 1.25%, and 1.09% respectively. Hence, a drastic decline in cell viability in these chambers, despite the presence of high glucose, is likely due to reduced oxygen levels below the critical value (Figure S5).

## Discussion

In this report, we have demonstrated a unique and simplified strategy to establish stable microfluidic gaseous gradients suitable for a variety of applications. Integration of real-time sensing capability and three-sided glass coating provides a versatile platform to study the effects of oxygen or any other gas on cell functions. Existing microfluidic oxygen gradient generation strategies utilize multilayer PDMS devices with gaseous inlets that are relatively more complex to fabricate and operate, and makes these devices susceptible to bubble formation. The proposed strategy utilizes a single layer device and a straightforward operating principle. Using two different pre-gassed tissue culture media solutions in gas-tight syringes, oxygen gradients of various spatial resolutions can be rapidly and conveniently established. The requirement of a separate gas layer or multiple gas inlets is thereby completely eliminated. Since pre-gassed media can be remotely prepared, stored, and transported, the platform proposed here can be utilized as a portable system for numerous applications even in resource limited settings. The integrated PtOEPK/PS sensor layer is highly sensitive to changes in oxygen concentrations. Polystyrene prevents leaching of the luminescent PtOEPK into the media, therefore any activity (whether cytotoxic or protein expression) can only be attributed to the varying oxygen conditions generated. Despite the fact that oxygen diffuses nearly twice as fast through PDMS than water^39^, the impermeability of glass coating and the thin membrane ensures constant net dissolved oxygen flowing through the aqueous buffer at any given point in time or location. The thin layer of PDMS on top of PtOEPK/PS layer allows instant diffusion of oxygen and consequent saturation of the dye, while enabling bonding of the top PDMS device to the substrate and a clear separation from cultured cells. Since the emission wavelength of PtOEPK is near the IR range (peak 760 nm) of the spectrum, the entire experiment (oxygen-gradient detection) can be conducted in a non-darkroom environment. The photostability of PtOEPK facilitates prolonged cell-based experiments without noticeable photobleaching or diminished detection sensitivity. Although, the characterization curves presented here verifies the gradient stability suggesting that integration of sensor layer is not necessary for cell based experiments. Thus, the gradient generation approach can also be utilized without the need for PtOEPK deposition step, if DO detection is not critical to the investigation. As previously mentioned, due to high diffusivity of oxygen in water and long residence time, the spatial resolution of DO gradient is diminished at low flow rates (less than 10 µL/min) in region III. However, oxygen gradient with high spatial resolution is maintained throughout regions I and II allowing for potential biological applications. The technique enables detection of dissolved oxygen with an enormous range of oxygen concentrations, only limited by the capability of the initial calibration electrode, and capacity of the fluid to hold oxygen. For this investigation, the range was between 0 mg/L to 19.9 mg/L. Adapting Henry’s Law, the corresponding percentage oxygen saturation in DI water ranged from 0% to 240%. Therefore, the current device can be used to explore the effects of extreme oxygen conditions on a plethora of microscopic aquatic species or flora, demonstrating its versatility and potential. The activity of marine heterotrophic bacterioplankton is highly dependent on partial CO_2_ pressure^43^, and *E.coli* metabolism is highly dependent on minute changes in oxygen concentration^44^. Therefore, with appropriate modifications in design, both aerobic and anaerobic analyses of bacteria under the effect of CO_2_ or O_2_ concentration gradients can be conveniently performed. Additionally, the split-channel devices have been previously utilized to generate gradients of multiple chemicals^45,46^. Similar strategies can be adapted to utilize pre-gassed solutions of two or more gases to generate more complex gradients extending versatility of the platform.

Effects of hypoxia in cancer cells serve as a major scientific paradox since it impedes survival, yet facilitates cancer progression^6,7^. Molecular oxygen is critical for post-translational folding of proteins in the ER^47^. In response to the high local content of misfolded proteins in the ER under hypoxic conditions, cancer cells can initiate the unfolded protein response (UPR) pathway^48^. The UPR activates a series of transmembrane proteins and transcription factors to rectify the misfolded proteins, despite the increasing ER stress levels^41,48^. Utilizing our technique, we were clearly able to demonstrate the rising ER stress in MDA-MB-468 cells with increasing oxidative stress at different time intervals across the single-outlet device, with a 4-fold increase from baseline in the hypoxic region after 6 hours. This enables a host of potential hypoxia activated responses such as cell motility, epithelial to mesenchymal transition etc. to be explored. Since unit concentrations are only generated in each outlet of the multiple-outlet device, the latter can serve the function of several specialized O_2_ incubators at once without requiring expensive infrastructure. Acquisition of drug resistance by tumor cells under hypoxic conditions can also be conveniently investigated. Due to continuous flow, local oxygen content in each outlet chamber will not be affected by the respiration of MCF-12A cells. It is noteworthy that the proposed platform is suitable for experiments involving adherent cells since cells in suspension will be flushed out by the continuous flow necessary to maintain the gradient, unless cells are physically trapped. The large width of the gradient chamber in region I (≈1.6 cm) in the single-outlet device provides shear stresses within the acceptable range that the cells can withstand regardless of the flow rate^49,50^.

One of the biggest limitations of PDMS-based microfluidic devices is the inability to conduct experiments with organic solvents. PDMS swells in contact with several organic solvents such as toluene significantly altering the channel dimensions. The proposed device not only enables the use of such solvents, characterization of parameters affecting the sol-gel reaction also allows optimization of glass coating thicknesses. Adhesion and diffusion of biomolecules within the hydrophobic PDMS surfaces can also be significantly inhibited by the glass coating.

## Methods

### Microfluidic device fabrication

The microfluidic chips were fabricated using established soft lithography procedures^51^. The initial design was prepared using AutoCAD software (Autodesk Inc) (Fig. 1b) and subsequently translated on a wafer with SU-8 photoresist using the direct write lithography system Dilase 250 (Kloe, France). SU-8 was first spin-coated onto the substrate, pre-baked, followed by direct write lithography, then subsequently post-baked and developed using the standardized procedures^52^. Liquid PDMS pre-polymer (Sylgard 184, Dow Corning, MI) was prepared by thoroughly mixing the base and curing agent (10:1 w/w) and poured onto the mold, degassed and thermally cured at 80 °C in an oven for 90 minutes^53,54^. PDMS devices were carefully cut with a scalpel and peeled and an 18-gauge flat end needle was used to make the inlets and outlets ports^54^. The devices were then sonicated in isopropyl alcohol for 30 s to remove the debris. Eventually, the channel side of devices were irradiated with oxygen plasma in a plasma asher (Plasma Etch, Carson City, NV) and irreversibly bonded to the PDMS/PtOEPK composite film (described in the next section) on a 1 × 3 inch microscope slide (Figure S2). Dimensions of the final device were dependent on the design-type, whether single-outlet (S1) or multiple-outlet (S1). This was decided through gradient profile data derived from several COMSOL simulations. The dimensions of the microfluidic serpentine channels in the single-outlet device were 250 (w) × 100 (h) µm. For the multiple-outlet device, the width of the serpentine channels was reduced to 200 μm and total length was reduced from 1.96 cm to 1.46 cm, effectively reducing residence time per flow rate. For all characterization purposes, 500 µm wide straight channels designs were used.

### Sensor layer formation

A modification of the standardized technique established by Nock *et al.* was used. Polystyrene pellets (MW ≈ 280,000, Sigma-Aldrich, St Lois, MO) were dissolved in toluene (Fisher Scientific, Waltham, MA) to yield a 7% w/w solution^19^. PtOEPK dye (Frontier Scientific, Logan, UT) was then added to form a stock solution at a concentration of 1 mg/ml^19^. 30 µL of PtOEPK/PS solution was pipetted onto a 1 × 3 inch microscopic glass slide and spin-coated at 1000 rpm for 30 s and left covered in dark at room temperature to dry for at least 2 hours. After drying, 0.2 gram of PDMS was placed on top of the PtOEPK/PS layer and spin-coated at 2500 rpm to form an approximately 1.5 µm thin layer of PDMS completely covering the dye. The resultant composite layer was then incubated at 40 °C in an oven for approximately 45 minutes (Figure S2).

### Sol-gel characterization and 3-sided glass coating

Sol-gel was prepared by mixing equal volumes of tetraethylorthosilicate (Sigma-Aldrich, St Lois, MO), methyltriethoxysilane (Sigma-Aldrich, St Lois, MO), ethanol and pH 4.5 deionized water and left for 24 hours to form a homogenous solution^37^. To establish a 3-sided glass coating, the sol-gel solution was pipetted through a plasma-treated PDMS device placed in close contact on a non-plasma treated glass slide. The device assembly was then placed on a pre-heated hotplate for the desired time and temperature and gently flushed with compressed air to remove excess unpolymerized silanol. The hotplate was turned off allowing the device to gradually cool down to room temperature. The curing of silanol oligomers (oxide bond formation) primarily occurs on the walls of the plasma-treated microfluidic channels as the hydroxyl groups are only present there. This preferentially creates a three-sided coating as no coating forms on the non-plasma treated bottom glass surface. To determine the optimal coating thickness, curing of silanol oligomers was conducted at varying temperatures and times (Table S1). After cooling, the coated PDMS was peeled off from the slide and plasma bonded to the sensor/PDMS layer (Figure S2). To confirm the glass-coating formation, sol-gel solution was mixed with fluorescent microspheres (1 µm diameter) (FluoSpheres ® Carboxylate, ThermoFisher Scientific Inc, Rockville, MD) prior to introduction into the microfluidic channels at 1:10 (dye: sol-gel) v/v concentration. The thickness of the coating on all three sides was measured through fluorescent images using the EVOS FL Auto microscope (Life Technologies). These results were later validated with the help of a confocal surface profilometer (LEXT OLS4100, Olympus, Japan). Both sets of images were taken by placing the glass coated PDMS devices upside down prior to bonding to the sensor composite layer. To test for leaching, 1 µM rhodamine B solution was infused through the 3 side-coated device assembly and compared with the uncoated controls for a 2-hour duration.

### Sensor layer calibration

Sensor-layer characterization and calibration was performed using the Olympus BX-51 fluorescence microscope (Japan). PtOEPK exhibits unique double absorption peaks at 398 nm and 590 nm and an emission peak at 760 nm in the near infrared region (NIR)^30,55^. A customized filter combination with absorption/emission filters at 570/760 nm and a 620 nm dichroic mirror was used for PtOEPK imaging.

For gaseous sensor characterization and to test the dynamic sensing ability, industrial grade oxygen and nitrogen gas (Roberts Oxygen, Rockville, MD) were blown into the straight channels periodically every 20 s. Air (21% O2) was blown though one of the channels constantly for reference. Images were recorded after reaching equilibrium and the respective intensities was analyzed using the Image Processing Toolbox in MATLAB (Mathworks, Natick, MA). Intensity was averaged over a region of interest and compared to the reference intensity (nitrogen saturated, ~0% O_2_). For dissolved oxygen (DO) measurement and characterization, DI water of varying oxygen concentrations were prepared. A customized oxygen and nitrogen delivery system was built and the gases were bubbled into capped glass bottles containing DI water for specific time periods. Bubbling nitrogen and oxygen for 40 minutes and 10 minutes respectively yielded DI water with 0.1 mg/L (0.003 mol/m^3^) and 19.9 mg/L (0.62 mol/m^3^) DO content respectively and intermediate DO solutions were obtained by mixing the two stock solutions appropriately. Detection of DO levels was done simultaneously, allowing us to determine exact bubbling periods for both gases. Sensor probe provides an “error” once DO levels go beyond 19.9 mg/L, thus, accuracy of the calibration was maintained throughout. Gassed water, kept in 1 ml syringes (BD Hamilton), were infused into the respective devices via 30-gauge blunt needle connected to the Tygon tubing (AAD04091, 0.01 “ID × 0.03” OD) using an automated syringe pump (Fusion 100, Chemyx, Stafford, TX). DO was measured using a polarographic oxygen meter (MW 600, Milwaukee Instruments, NC, USA). Signal intensity, I, with respect to O_2_ concentration is quantified by the following Stern-Volmer relationships^40,56–58^:

1 $${\rm{Gaseous}}\,{\rm{oxygen}}:\,\,\,\,\,\,\,\,I/{I}_{o}=\{{f}_{1}/(1+{{K}_{G}}^{sv}p{O}_{2})\}+{f}_{2}$$

2 $$\mathrm{Dissolved\; oxygen}:\,\,\,\,\,{I}_{o}/I=1+{{K}_{S}}^{sv}[{O}_{2}]$$

where K_S_^sv^ and K_G_^sv^ are the Stern-Volmer constants for solution and gas respectively, I_o_ represents intensity at 0% oxygen (100% nitrogen), [O_2_] is oxygen concentration and pO_2_ is partial pressure of gaseous O_2;_ f_1_ is the portion dye molecules being quenched and f_2_ being unquenched portion. Plots of relative intensities against DO concentrations and gaseous partial pressure of O_2_ respectively were extrapolated to validate the Stern-Volmer relationship. Plots of intensity changes during periodic introduction of oxygen and nitrogen (both gaseous and dissolved) were also generated. This data was subsequently used as reference for the intensity variation or changes during the gradient formation in the final fabricated devices.

### Gradient formation and detection

Initially, linear flow rates and resulting gradient formation were determined using COMSOL Multiphysics software. These derived conditions were subsequently implemented in the experiments. O_2_-rich and O_2_-depleted deionized water was passed at constant flow rates through the microfluidic channels of the final fabricated devices. Diffusion and oxygen detection was monitored using time-lapse epifluorescent microscopy using the Olympus BX-51 fluorescence microscope. The recorded video was imported onto MATLAB and subsequently fragmented into frames. Intensity plots were extracted and analyzed across the desired frames at different flow rates using Image Processing Toolbox in MATLAB. The respective plots were converted to concentration plots using the previous calibration data, yielding a function curve for each flow rate. Triplicate experiments, along with calibration before each, were conducted and average function plots for respective flow rate were obtained.

### Cell culture, evaluation of endoplasmic reticulum stress and viability under hypoxia

The evaluation of ER stress and cellular viability under oxidative stress were conducted using MDA-MB-468 cells and MCF-12A respectively (ATCC, Manassas, VA). Both cell-types were cultured initially in T25 flasks under standard 5% CO_2_. For initial control, the MDA-MB-468 and MCF-12A cells were seeded in 2 separate 24-well plates, one for incubation under normal oxygen conditions (21% O_2_ and 5% CO2) and the other was incubated in a hypoxic chamber (95% N_2_ and 5% CO_2_) (Billups-Rothenberg, CA). Both were incubated for 6 hours. The channels of both devices were first treated with 100 µg/ml fibronectin solution in PBS (F2006, Sigma-Aldrich Co., St. Louis, MO) overnight before introducing cells. Devices were then subsequently washed with PBS (phosphate buffer saline) and cell suspensions were then introduced. MDA-MB-468 cells (2500 cells/µL) were introduced into the region I, ensuring a packed and uniform distribution of cells throughout. MCF-12A cells (1000 cells/μL) were introduced into each of the chambers of the multiple-outlet device. Prior to introduction, the MCF-12A cells were treated with 10 µM Cell Tracker Green CMFDA dye (ThermoFisher Scientific, Waltham, MA, USA) for 30 minutes for live-cell analysis.

For the ER stress evaluation, regular (21% oxygen) and deoxygenated DMEM media (0%), both containing thioflavin T (5 μg/ml), were introduced simultaneously through the two inlets of the gradient generator at a constant flow-rate of 1 µL.min^−1^ for 6 hours in one device. Another single-outlet device was used to observe the endogenous ER stress levels in MDA-MB-468 cells by using normal media in both inlets, thus acting as control. Baseline ER stress was recorded prior to starting the experiment. Time-lapse images were acquired every 30 minutes to monitor the fluorescence profile using EVOS-FL Auto Cell Imaging System (ThermoFisher Scientific, Waltham, MA, USA). Image analysis of cells was conducted using ImageJ (NIH, Bethesda, MD) and MATLAB. For the viability experiment, regular and deoxygenated DMEM F-12 media were simultaneously infused for 6 hours and time-lapse images taken every 30 minutes. 10 µM Cell Tracker Red CMTPX dye and 8 µM Hoechst stain were used for live cell-staining in the control. The 0% oxygen media were created by bubbling nitrogen gas into 1 ml of DMEM and DMEM F-12 media for approximately one hour. 15 µM HEPES solution was added to both in order to maintain pH of the media coherent with 5% CO_2_ before adding to the cell culture. Viability analysis was performed based on the absolute initial and final cell count within each chamber. However, initial number of cells within individual chambers was not identical. Therefore, to appropriately demonstrate the relative decline in cell viability across different chambers, normalization was performed. The resulting cell count at each interval was calculated as a percentage of the initial count at “T = 0”.

### Statistical analysis

For most experiments, one-tailed *t test* was performed to determine statistical significance of the data. Functional analysis was conducted in MATLAB to compare COMSOL and experimental data plots for gradient detection. Unless explicitly stated, all experiments were performed in triplicate. Error bars represent standard deviation of the mean. One-way ANOVA was used to analyze means of experimentally derived gradients across different flow rates in the gradient chamber with the corresponding COMSOL data.

### Data availability

All data generated or analyzed during this study are included in this article (and Supplementary Information). Additional information can be obtained from the corresponding author on reasonable request.

## Electronic supplementary material

Supplementary Information
