# Supplementary material for: Rapid Generation and Detection of Biomimetic Oxygen Concentration Gradients In Vitro
Source: Sci Rep. 2017 Oct 18;7:13487. doi: 10.1038/s41598-017-13886-z (PMC5647399; doi:10.1038/s41598-017-13886-z)
Supplement: Supplementary file 1 — Supplementary Information [file 41598_2017_13886_MOESM1_ESM.pdf]

Rapid Generation and Detection of Biomimetic Oxygen Concentration Gradients In Vitro

Md. Daud H. Khan[1], Steven A. Roberts[1], John Robert Cressman[2], Nitin Agrawal[1*]

[1]Bioengineering Department, George Mason University, Fairfax 22030, USA

[2]School of Physics, George Mason University, Fairfax 22030, USA

*Correspondence and requests for materials: Nitin Agrawal (nagrawa2@gmu.edu)

	Temperature (^{0}C)				
**Time (s)**	**60**	**70**	**80**	**90**	**100**
**20**	3.56 ±1.98 µm	4.77 ±1.87 µm	5.06 ± 1.33 µm	5.84 ± 1.62 µm	6.02 ± 0.94 µm
**40**	4.25 ±1.52 µm	6.86 ±0.58 µm	6.88 ± 0.78 µm	7.43 ± 0.41 µm	7.73 ± 1.14 µm
**60**	8.65 ±1.02 µm	9.01± 1.99 µm	9.47 ± 1.21 µm	9.96 ± 0.98 µm	11.25 ± 0.47 µm
**80**	12.52± 0.86 µm	12.47 ± 2.55 µm	13.33 ± 0.65 µm	13.87 ± 1.68 µm	14.42 ± 1.19 µm
**100**	13.2 ± 2.05 µm	13.42 ± 1.74 µm	14.07 ± 1.87 µm	14.68 ± 2.41 µm	15.99 ± 2.65 µm

Table S1: Average glass-coating thickness across different reaction temperatures and times. Green cells represent optimal conditions that provide coatings with no unpolymerized spaces and minimal cracks.

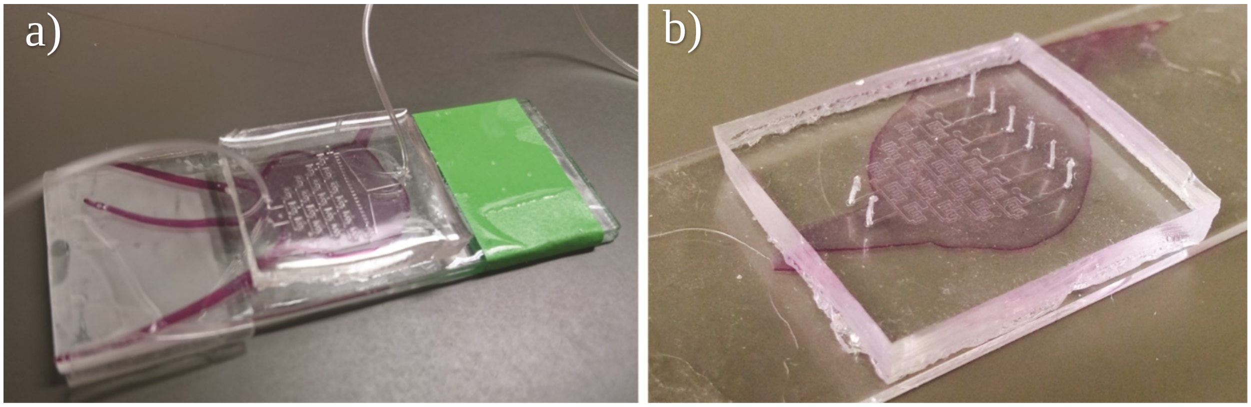

Figure S1: (a) Fabricated single-outlet device with channel dimensions of 250 µm (w) x 100 µm (h) and the length of each serpentine segment is approximately 7.6 mm. The blocks, separated by 187.5 µm gap, are placed as support structures to prevent the top PDMS surface from collapsing. **(b)** Fabricated multiple-outlet device with channel dimensions of 200 µm (w) x 100 µm and the same length of each serpentine segment as the single-outlet device.

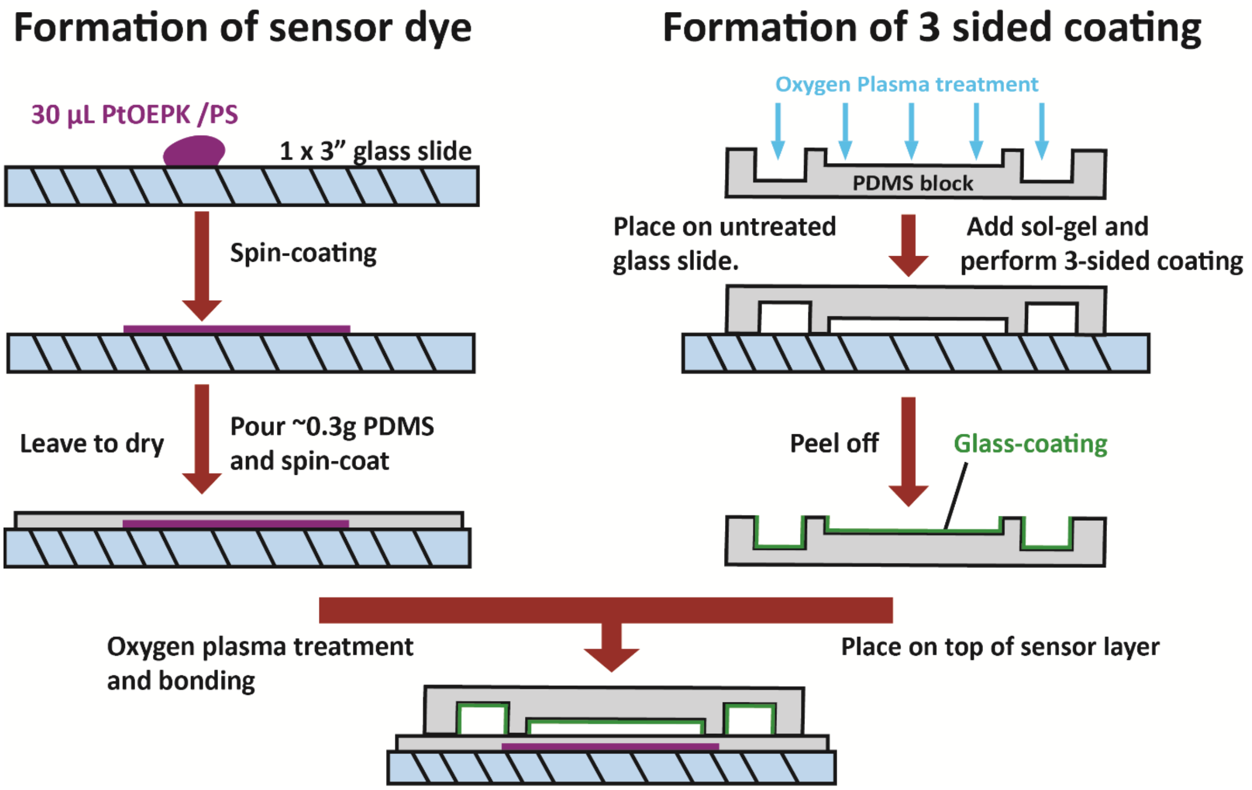

Figure S2: Schematic showing the steps of device fabrication process.

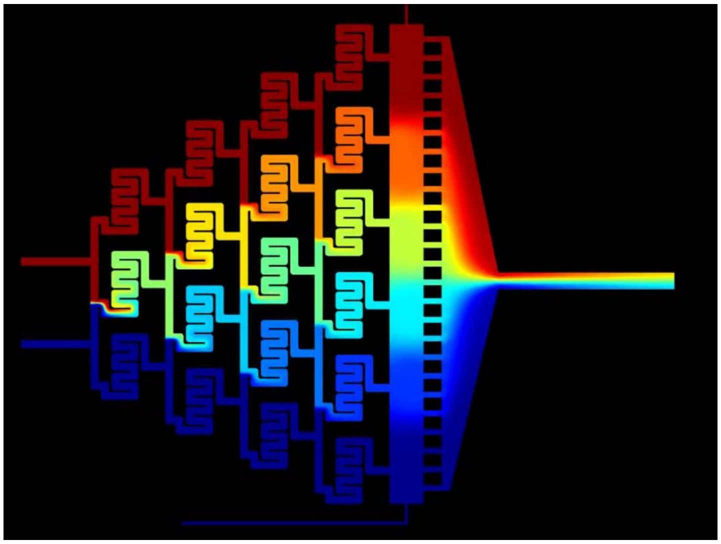

Figure S3: COMSOL simulated oxygen gradient profile in single-outlet device at 10 nL/min flow rate. Though overall gradient across the gradient chamber is the same throughout, the oxygen profile tends to equilibrate by the time it approaches the outlet due to very long residence time of 49 minutes.

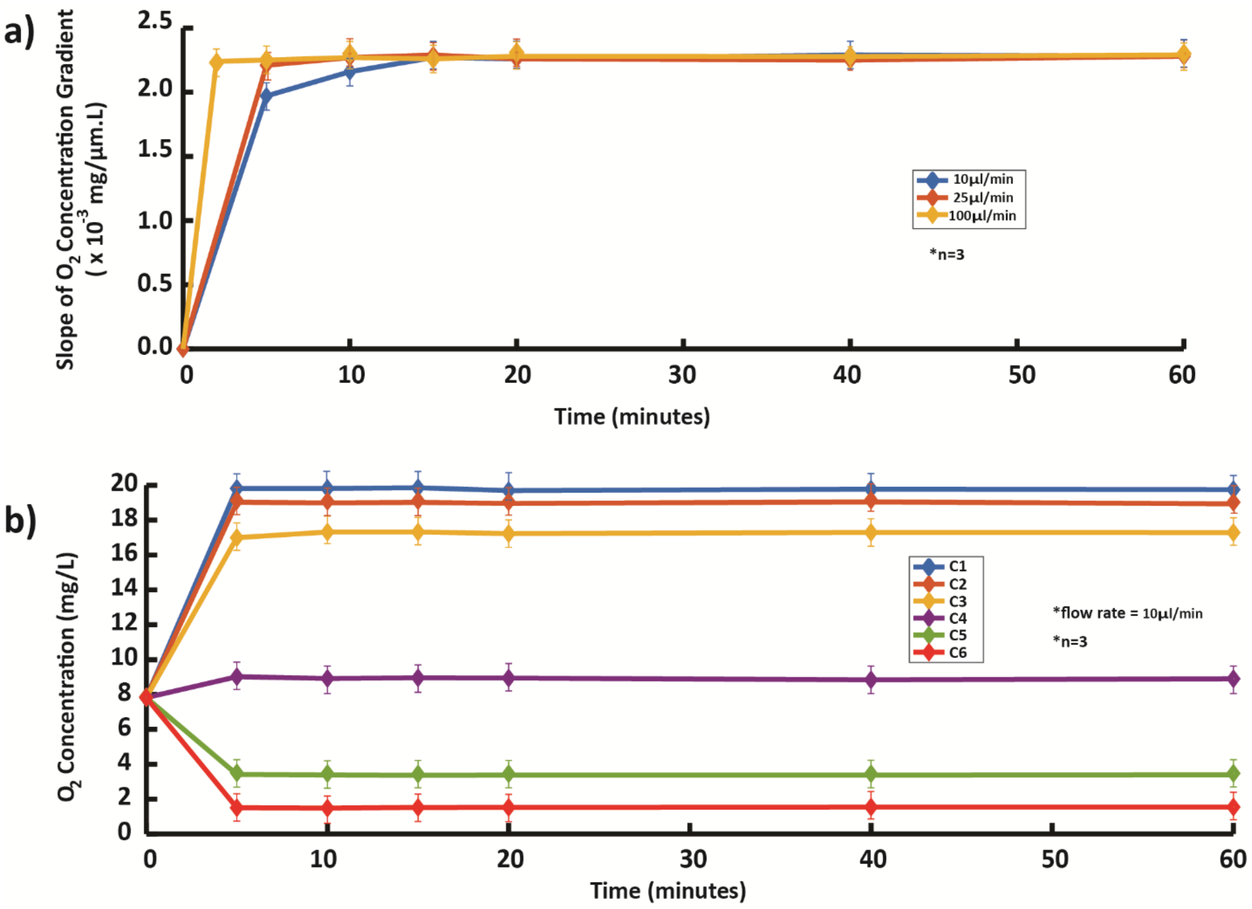

Figure S4: (a) Plot illustrating the slope of gradient across gradient chamber in the single-outlet device over time at different flow rates. This shows the time required to formed respective gradients and also that gradients are stable over lengthy time periods. **(b)** Plot illustrating the stability of unit concentrations of oxygen in each of the respective chamber or outlet inside the multiple-outlet device, also exhibiting stability over a 1 hour time period.

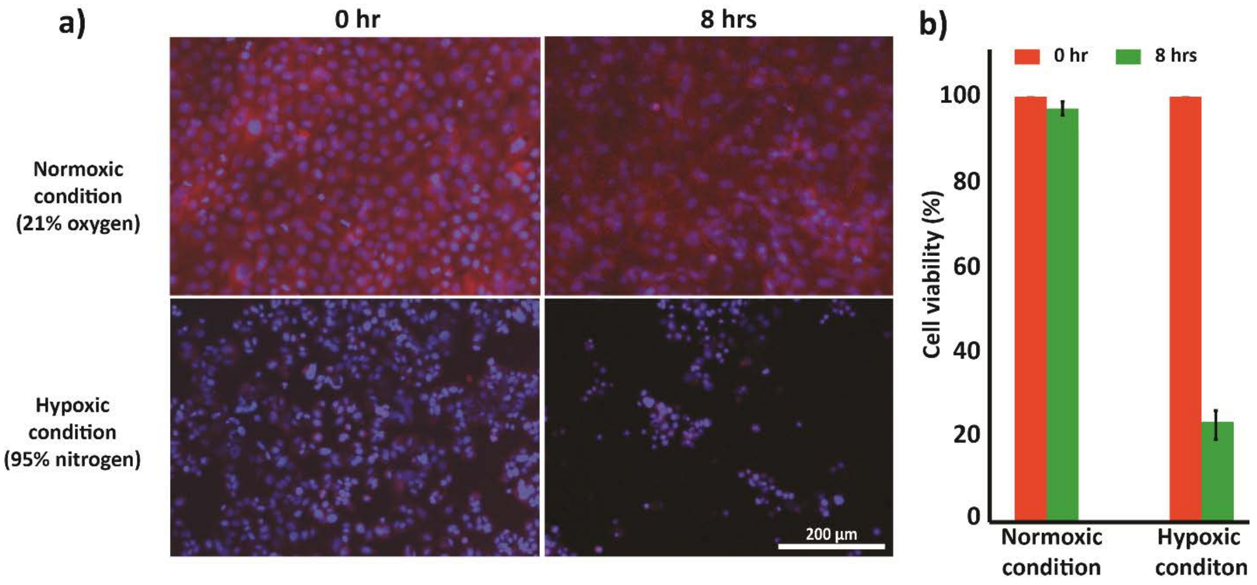

Figure S5: Viability analysis of MCF-12A cells in a 96-well plate for normal and hypoxic condition **(a)** Micrographs of cells stained with CMTPX for cytoplasm and Hoechst for nuclei. **(b)** Quantitative analysis of cell viability showing ~80% decrease in cell viability under hypoxic condition.

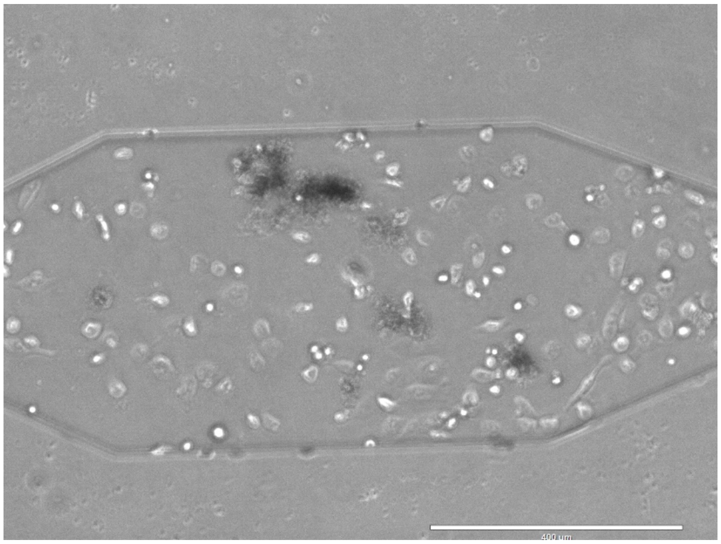

Figure S6: Brightfield image of MCF-12A cells cultured within one of the chambers or outlets of the multiple-outlet device under normal conditions.
